# 1319. Assessment of Spectrum Score-Based Antibiotic De-Escalation in Patients with Nosocomial Pneumonia

**DOI:** 10.1093/ofid/ofab466.1511

**Published:** 2021-12-04

**Authors:** Daniel T Ilges, Elizabeth Neuner, Tamara Krekel, David J Ritchie, Nicholas B Hampton, Scott Micek

**Affiliations:** 1 Barnes-Jewish Hospital, St. Louis, Missouri; 2 BJC Healthcare, St. Louis, Missouri; 3 Barnes Jewish Hospital, St Louis, Missouri

## Abstract

**Background:**

Hospital-acquired and ventilator-associated pneumonia (HAP/VAP) cause significant morbidity and mortality. Guidelines recommend broad-spectrum empiric antibiotic therapy, including treatment for *Pseudomonas aeruginosa* (PSAR) and methicillin-resistant *Staphylococcus aureus* (MRSA), followed by de-escalation (DE). This study sought to assess the impact of DE on treatment failure.

**Methods:**

This single-center retrospective cohort study screened all adult patients with a discharge diagnosis code for pneumonia from 2016–2019. Patients were enrolled if they met pre-defined criteria for HAP/VAP ≥48 hours after admission. Date of pneumonia diagnosis was defined as day 0. Spectrum scores were calculated, and DE was defined as a score reduction on day 3 versus day 1. Patients with DE were compared to patients with no de-escalation (NDE). Data were compared using chi-square, Mann-Whitney U, or T-tests. The primary outcome was composite treatment failure, defined as all-cause mortality or re-admission for pneumonia within 30 days of diagnosis, analyzed using a Cox proportional hazards analysis to control for confounding variables.

Figure 1. Study Schematic

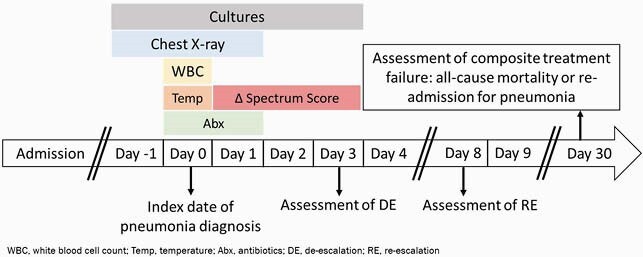

Table 1. Spectrum Score Assignment

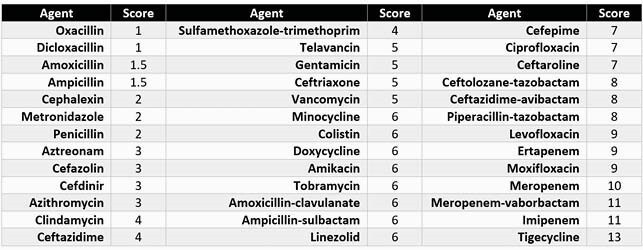

**Results:**

Of 11860 admissions screened, 1812 unique patient-admissions were included (1102 HAP, 710 VAP). Fewer patients received DE (876 DE vs. 1026 NDE). Groups were well-matched at baseline, although more patients receiving DE had respiratory cultures ordered (56.6% vs. 50.6%, P=0.011). Patients receiving DE experienced a 65% and 44% reduction in anti-MRSA and anti-PSAR therapies by day 3, respectively. There was no difference in composite treatment failure (35.0% DE vs. 33.8% NDE, P=0.604). DE was not associated with treatment failure on Cox multivariate regression analysis (HR 1.13, 95% CI 0.97-1.33, P=0.149). Patients receiving DE had fewer antimicrobial days (median 9 vs. 11, P< 0.0001), episodes of *Clostridioides difficile* (2.2% vs. 3.8%, P=0.046), and days of hospitalization (median 20 vs. 22 days, P=0.006).

Figure 2: Median Spectrum Scores (SS) Days 0 to 28

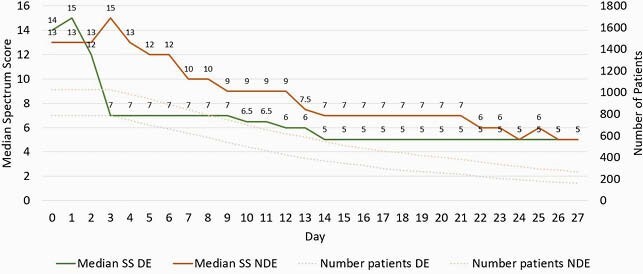

Table 2: Cox Regression Analysis

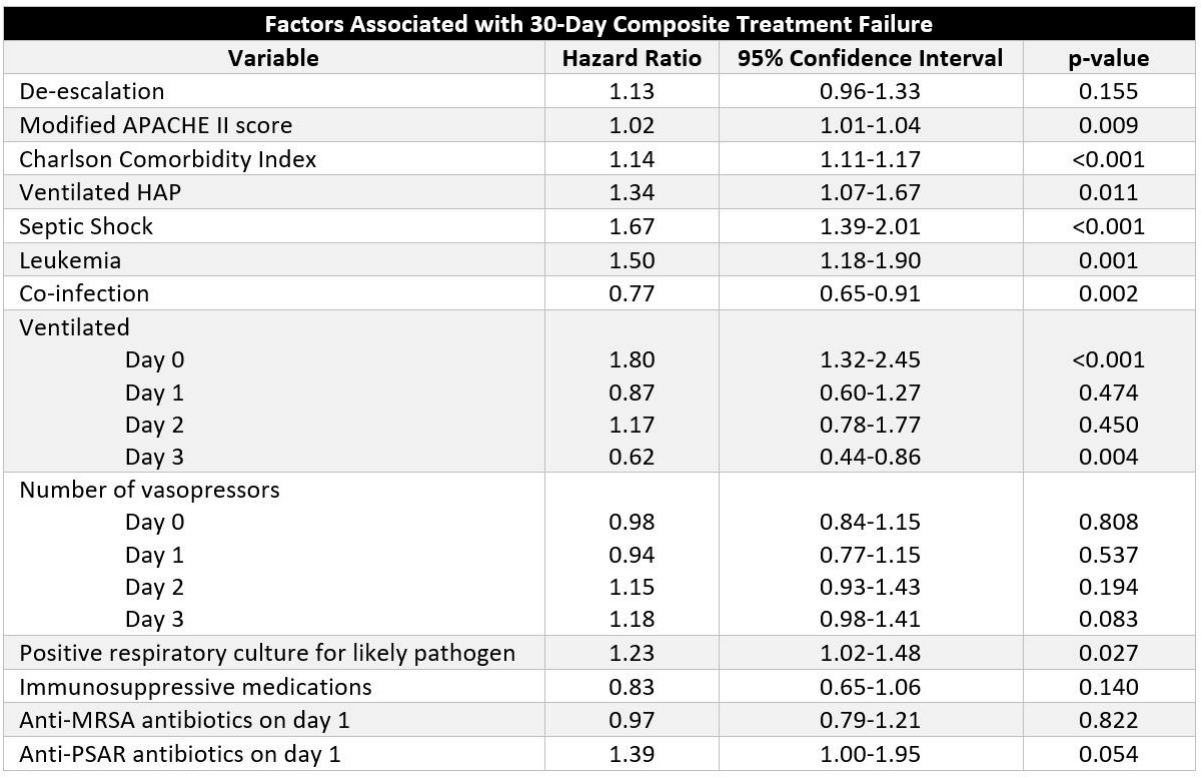

**Conclusion:**

DE and NDE resulted in similar rates of composite treatment failure at 30 days; however, DE was associated with fewer antimicrobial days, episodes of *C. difficile*, and days of hospitalization. Spectrum scores can objectively identify DE, but further studies are needed to fully understand their utility in this context.

**Disclosures:**

**Tamara Krekel, PharmD, BCPS, BCIDP**, **Merck** (Speaker’s Bureau) **David J. Ritchie, PharmD, BCPS (AQ-ID**), **AbbVie** (Speaker’s Bureau)**Merck** (Speaker’s Bureau)

